# 

**Published:** 1903-04-11

**Authors:** 


### NOTICE TO CORRESPONDENTS.

All MSS., Letters, Books for Review, and other matters intended for the Editor should be addressed The Editor, "The Hospital," The Hospital Buildings, 28 & 29 Southampton Street, Strand, W.C.

The Editor cannot undertake to return rejected MSS., even when accompanied by stamped directed envelope.

**Notice to Advertisers and Subscribers.**

All Advertisements, Orders for Copies of The Hospital, and Business Communications should be addressed to **THE MANAGER, (Not to the Editor),** The Hospital, 28 & 29 Southampton Street, Strand, London, W.C.

The Cost of Subscription, post free, is as follows.—Per week, 3½d. ; per quarter, 3s. 9d.; per half-year, 7s. 6d.; per annum, 15s. Foreign and Colonial :—Per annum, 19s. 6d., payable, in all cases, in advance.

**NOTICE.—Vol. XXXIII. of "The Hospital" (October 1902 to March 1903) in handsome cloth binding, will shortly be on sale at the office of this Journal, price 7s. 6d. Cases for binding the Numbers contained in this Volume 1s. 6d. Index to Vol. XXXIII., 2½d., post free.**

**The monthly part for April is now ready. Price 6d., by post 8d.**

### The Student of Medicine.

### APPOINTMENTS.

**Barclay J. Baron**, M.B., C.M.Edin., appointed Honorary Consulting Physician to the Throat and Nose Department of the Bristol General Hospital. **C. Blue**, M.B., B.Ch.R.U.I., appointed Medical Officer for the Skelton District of the Guisborough Union. **Walter Chapman**, M.B., Ch.B., F.R.C.S., L.R.C.P., appointed Surgeon to the Birmingham Corporation Waterworks and Hospital in Elan Valley. **R. Dawe**, M.D.Lond., B.Sc., appointed Assistant Medical Officer to the London Fever Hospital. **John Shields Fairbairn**, M.B., B.Ch.Oxon., F.R.C.S.Eng., M.R.C.P.Lond., appointed a Physician to Out Patients of the British Lying-in Hospital, Endell Street. **John Lacy Firth**, M.D., M.S.Lond., appointed Physician to the Throat and Nose Department of the Bristol General Hospital. **George Foggin**, B.A.Lond., L.R.C.P.&S.Edin., etc., appointed Principal Medical Officer to the Newcastle-upon-Tyne School Board. **Nathan Hannah**, L.R.C.P.Edin., L.F.P.S.Glas., appointed Certifying Factory Surgeon for the Ashton-in-Makerfield District in the county of Lancaster. **F. P. Hearder**, M.D.Edin., appointed Senior Assistant Medical Officer to the North Riding Asylum, York. **Hedley Hill**, M.D.Brux., M.R.C.S., L.R.C.P.Lond., appointed Assistant Anæsthetist to the Bristol General Hospital. **Charles Wm. Low**, M.B.Durh., D.P.H., reappointed Medical Officer to the Stowmarket Urban District Council. **F. C. Whitmore**, M.R.C.S., appointed Deputy Medical Officer for the Workhouse, and the No. 1 District of Thornbury Union. **Wm. Yeates**, L.R.C.P.Edin., L.R.C.S.Edin., L.F.P.&S.Glasg., appointed Medical Officer to the Heworth District of the Gateshead Union.

28 & 29 Southampton Street, Strand, London, W.C.

### SUCCESS.

Efficiency is the keynote of success in any profession, but especially is it so in the Nursing Profession. When one recollects the enormous strides that have been made in this important calling within the last fifty years, the truth of this statement is fully borne out. To attain efficiency, and its accompanying success, it is essential that the nurse should keep abreast with all that is new and up-to-date in the many branches of her profession. As a means to this end, the works of recognised and competent teachers will be found of the greatest assistance.

It is the object of The Scientific Press to supply nurses with the best and latest manuals on Nursing and Allied Subjects, and we append a selected list of some of the most valuable books.

**NURSING.** — **THE NURSING PROFESSION: HOW AND WHERE TO TRAIN.**
By Sir Henry Burdett, K.C.B.
Price 2/- net.; post free, 2/4.

**A HANDBOOK FOR NURSES.**
By J. K. Watson, M.D., M.B., C.M.
New Edition. Price 5/- net.; post free, 5/4.

**NURSING: ITS THEORY AND PRACTICE.**
By Percy G. Lewis, M.D., M.R.C.S., L.S.A.
New Edition. Price 3/6 post free.

**SURGICAL WARD-WORK.** — **SURGICAL WARD-WORK AND NURSING.**
By Alex. Miles, M.D.Edin.
Second Edition. Price 3/6 net.; post free, 3/10.

**ANATOMY.** — **ELEMENTARY ANATOMY AND SURGERY FOR NURSES.**
By Wm. McAdam Eccles, M.S.
Price 2/6 post free.

**PHYSIOLOGY.** — **ELEMENTARY PHYSIOLOGY FOR NURSES.**

By C. F. Marshall, M.D., B.Sc., F.R.C.S.

Price 2/- post free.

**MASSAGE.** — **ART OF MASSAGE.**

By Mrs. A. Creighton Hale.

Second Edition. 6/- Post free.

**MIDWIFERY.** — **A PRACTICAL HANDBOOK OF MIDWIFERY.**
By Francis W. Haultain, M.D.
New and Revised Edition. 6/- net.; post free, 6/3.

**THE NURSE'S DICTIONARY OF MEDICAL TERMS AND NURSING TREATMENT.**
Compiled for the use of Nurses. By Honnor Morten.
Fourth and Revised Edition. Demy 16mo. (suitable for the apron pocket), bound in cloth boards, 160 pp., 2/- post free; in handsome leather, gilt, 2/6 net; 2/8 post free.

*A Complete Catalogue will be forwarded Post Free on application.*

THE SCIENTIFIC PRESS, LIMITED, 28 & 29 Southampton Street, Strand, London, W.C.

